# Knockdown of lncRNA CCAT1 Inhibits the Progression of Colorectal Cancer via hsa-miR-4679 Mediating the Downregulation of GNG10

**DOI:** 10.1155/2021/8930813

**Published:** 2021-12-30

**Authors:** Ning Wang, Jun Li, Ju He, Yong-Guang Jing, Wei-dong Zhao, Wen-jin Yu, Jing Wang

**Affiliations:** ^1^Department of Surgery, School of Clinical Medicine, Dali University, Dali, Yunnan 671000, China; ^2^Department of General Surgery, The First Affiliated Hospital of Dali University, Dali University, Dali, Yunnan 671000, China; ^3^Department of Human Anatomy, School of Basic Medical Sciences, Dali University, Dali, Yunnan 671000, China; ^4^Laboratory Department, School of Clinical Medicine, Dali University, Dali, Yunnan 671000, China

## Abstract

Great concerns have raised crucial roles of long noncoding RNAs (lncRNAs) on colorectal cancer progression due to the increasing number of studies in cancer development. Previous studies reveal that lncRNA CCAT1 plays an important role in the progression of a variety of cancers. However, the role of lncRNA CCAT1 in colorectal cancer is still unclear. In this study, we found that in both colorectal tissues and cell lines the level of lncRNA CCAT1 was increased. Downregulation of lncRNA CCAT1 inhibited the proliferation, migration, and invasion of colorectal cell lines and promoted apoptosis. We then found that hsa-miR-4679 could bind to lncRNA CCAT1 directly, and with further functional analyses, we confirmed that lncRNA CCAT1 sponged hsa-miR-4679 to promote the progression of colorectal cancer. Next, we found that hsa-miR-4679 was directly bound to 3′UTR of GNG10 (guanine nucleotide-binding protein, gamma 10). GNG10 overexpression promoted the progression of colorectal cancer, and this phenotype could be reversed by miR-4679 mimics. At last, we knocked down CCAT1 *in vivo* and found that sh-CCAT1 reduced the tumor size and the number of proliferating cells. In summary, our findings revealed that lncRNA CCAT1 facilitated colorectal cancer progression via the hsa-miR-4679/GNG10 axis and provided new potential therapeutic targets for colorectal cancer.

## 1. Introduction

Colorectal cancer (CRC), the fourth most deadly cancer in the world, resulting in 900 000 deaths annually, is caused by hereditary and environmental risk factors, including aging, dietary habits of developing countries, obesity, lack of physical exercise, and smoking [1, 2]. Recently, great concerns have been arisen by the increasing number of colorectal cancer patients younger than 50 years, especially rectal cancer and distal colon cancer [3–5]. Nowadays, colorectal cancer is normally treated with long-course chemoradiotherapy or short-course radiotherapy, combining the total mesorectal excision [6]. However, CRC patients respond to the common therapy differently, which may be due to the diverse causes on the cellular and molecular levels [7–9]. Multiple molecules regulated the initiation and progression of CRC as a network. However, the mechanisms underlying the initiation and progression of CRC are still poorly understood.

Long noncoding RNAs (lncRNAs), which are longer than 200 nucleotides without protein-coding ability, are involved in multiple processes in CRC cell proliferation, migration, invasion, and apoptosis [10]. lncRNAs regulate the transcription process of a variety of proteins related to CRC via binding to microRNAs in the competitive endogenous RNA (ceRNA) manner [11–13]. Long noncoding RNA colon cancer-associated transcript-1 (CCAT1), which contains 2628 nucleotides, is firstly identified in colon cancer. CCAT1 plays crucial roles in multiple cancers, including gastric carcinoma, colon cancer, gallbladder cancer, and hepatocellular carcinoma, during the proliferation, migration, invasion, apoptosis, and drug resistance processes [14–19]. There are accumulating studies revealing the affected downstream pathways of CCAT1 in CRC patients, such as miR-181a-5p, miR-181b-5p, and miR-218 and the interaction of MYC promoter and its enhancers [20–23]. It is obvious that a variety of molecules is involved in the development of CRC and forms a pathway network to regulate the progression of CRC. However, the downstream pathways are still poorly understood.

Here, we reported that in both CRC tissues and CRC cell lines CCAT1 was upregulated, and knockdown of CCAT1 inhibited the CRC progression by inhibiting the proliferation, migration, and invasion and promoted cell apoptosis. Next, we found that hsa-miR-4679 was downregulated in CRC and directly bound to CCAT1 in an AGO2 manner. sh-CCAT1 could efficiently reverse the promoted CRC progression by hsa-miR-4679 inhibitors. Furthermore, we identified GNG10 as the protein effector of the CCAT1/hsa-miR-4679 axis. At last, we performed xenograft tumorigenesis experiments *in vivo* and found that sh-CCAT1 could significantly inhibit the tumorigenesis in vivo by promoting the proliferation and inhibiting the apoptosis through upregulation of hsa-miR-4679 and downregulation of GNG10. In summary, we found that lncRNA CCAT1 promoted the progression of CRC via hsa-miR-4679 mediating the expression of GNG10.

## 2. Materials and Methods

### 2.1. Clinic Samples

20 pairs of patient CRC tissues and corresponding adjacent control samples were collected according to the guidelines of the Ethics Committee of Dali University.

### 2.2. Cell Culture and Transfection

FHC, a human colon immortalized cell line, was cultured in DMEM : F12 medium with 10 mM HEPES, 10 ng/ml cholera toxin, 0.005 mg/ml insulin, 0.005 mg/ml transferrin, 100 ng/ml hydrocortisone, 20 ng/ml human recombinant EGF (BMS320, Thermo Fisher, USA), and 10% fetal bovine serum (10099, Thermo Fisher, USA). HCT-116, a CRC cell line, was cultured in RPMI-1640 containing 10% fetal bovine serum (10099, Thermo Fisher, USA). HT-29, a CRC cell line, was cultured in RPMI-1640 containing 10% FBS (10099, Thermo Fisher, USA). All cell lines were cultured under 37°C with 5% CO_2_ in the incubator. To silence CCAT1, the short hairpin RNA (shRNA) sequence used for targeting CCAT1 was 5′-ACCCCATTCCATTCATTTCTCTTTCCTATTCAAGAGATAGGAAAGAGAAATGAATGGAATGGTTTTTTG-3′. The shRNA sequence used for targeting CCAT1 was subcloned into pSuper vector. The miRNA inhibitor was small single-stranded RNA molecules, which were synthesized via chemical methods. The miRNA inhibitor could competitively bind to the targeting miRNA via complementarity to binding sequence, acting like a molecule sponge to soak up miRNA. All the chemically modified small molecules were synthesized in GenePharma (Shanghai, China). Lipofectamine 3000 was used for the transfection of CRC cells for in vitro assays. 48 hours after cell transfection, cells were harvested for the downstream analyses.

### 2.3. Total RNA Extraction and Quantitative PCR

Total RNA was extracted using TRIzol™ Reagent (15596026, Thermo Fisher, USA) according to the manufacturer's guidelines. SYBR Green PCR Master Mix (4368577, Thermo Fisher, USA) was used for quantitative PCR to detect the expression of CCAT1, hsa-miR-4679, and GNG10. The expression levels of CCAT1, hsa-miR-4679, and GNG10 were normalized using GAPDH with the 2^-*ΔΔ*Ct^ method.

Primers list is as follows: MystiCq® microRNA qPCR Assay Primer (MIRAP01193, Merck, USA); CCAT1 forward: TTTATGCTTGAGCCTTGA 3′ and CCAT1 reverse: CTTGCCTGAAATACTTGC 3′; GAPDH forward: 5′-TGTGGGCATCAATGGATTTGG-3′ and GAPDH reverse: 5′-GGAGAGGGAAGTTACGGAACA-3′; GNG10 forward: 5′-TGGTAGAGCAGCTCAAGTTGG-3′ and GNG10 reverse: 5′-CAGCAAACTTCTCTCCTAGAGTC-3′.

### 2.4. EdU Detection

For the assessment of the proliferation ability of CRC cells, Click-iT™ EdU Imaging Kits (C10337, Thermo Fisher, USA) were used to label proliferated cells with EdU according to the user guideline.

### 2.5. Western Blotting

Lysate from cell culture was prepared using precooled NP-40 lysis buffer. Tissues were dissected and homogenized on ice and then treated with precooled NP-40 lysis buffer. Protein samples were boiled for denaturing and loaded on the gel for protein separation. Then, the proteins were transferred into PVDF membranes, incubated with primary and secondary antibody solution, and detected using ECL detection reagents. Antibodies used in this paper were as shown: Sox2 (1 : 1000, 3728S, Cell Signaling Technology, USA); Ki-67 (1 : 1000, MA5-14520, Thermo Fisher, USA); PCNA (1 : 1000, ab92552, Abcam, USA); GNG10 (1 : 500, LS-C409958, Lifespan Biosciences, USA); cleaved caspase-3 (1 : 1000, ab2302, Abcam, USA); Bax (1 : 1000, MA5-35342, Thermo Fisher, USA); Bcl-2 (1 : 1000, AF810, R&D System, USA); and GAPDH (1 : 1500, ab8243, Abcam, USA).

### 2.6. Cell Migration and Invasion Assays

HT-29 cells were transfected with sh-CCAT1 and/or hsa-miR-4679 inhibitor or hsa-miR-4679 mimics and/or GNG10 overexpression plasmids. After 24 hours, cells were digested, washed with PBS, and resuspended with serum-free DMEM. 3 × 10^5^ cells were seeded onto the upper chambers of transwell with polycarbonate membrane insert (3402, Corning, USA), and 800 *μ*l culture medium with 10% fetal bovine serum was added at the bottom. Cells were cultured under 37°C with 5% CO_2_ in the incubator for 24 hours, and then, the bottom migrated cells were fixed, stained with trypan blue, and imaged under a microscope. For the evaluation of invasion ability, the upper chamber was coated with Matrigel before cell seeding.

### 2.7. Apoptosis Rate Measurement

The apoptosis rate of HT-29 cells with certain treatments was assessed using the flow cytometer. Cells were digested, washed with precooled PBS, resuspended, and incubated with Annexin V-FITC and PI at room temperature for 10 min. Annexin V+/PI- cells are early-stage apoptosis cells, and Annexin V+/PI+ cells are late-stage apoptosis cells.

### 2.8. Luciferase Activity Assay

The target fragments and corresponding mutant fragments were subcloned into the luciferase reporter vector. Then, the designed luciferase reporter vectors were cotransfected with hsa-miR-4679. The luciferase activities were evaluated with Promega dual-luciferase reporter assay system according to the user manual.

### 2.9. RNA Immunoprecipitation Assay

HT-29 cells transfected with hsa-miR-4679 mimics or controls were lysed and incubated with anti-AGO2 or anti-IgG and protein A/G beads. Relative expression of CCAT1 was evaluated using qPCR.

### 2.10. Nude Mouse Tumorigenesis

All experiments involving animals were done according to the guidelines of the Ethics Committee of Dali University. Six-week-old, SPF-grade BALB/C nude mice were divided into 2 groups: control and sh-CCAT1. 10^5^ HT-29 cells were subcutaneously injected into the nude mice. Three weeks after the injection, mice were anesthetized and sacrificed for tumor collection. Tumors were measured for the volume calculation.

### 2.11. H&E Staining and Immunohistochemistry Staining

Tumor tissues were collected, fixed with precooled 4% PFA, dehydrated, and sectioned. Sample sections were stained with hematoxylin-eosin or Ki-67 primary antibody Ki-67 (1 : 800, MA5-14520, Thermo Fisher, USA).

### 2.12. Statistical Analysis

All statistical analyses were performed with GraphPad Prism 8.0. All data were presented as the mean ± standard error with at least 3 independent repeats. The differences were analyzed under a two-tailed Student's *t*-test. *p* value less than 0.05 was considered as the significant difference.

## 3. Results

### 3.1. Knockdown of lncRNA CCAT1 Inhibited the Progression of Colorectal Cancer Progression

**Figure 1:**
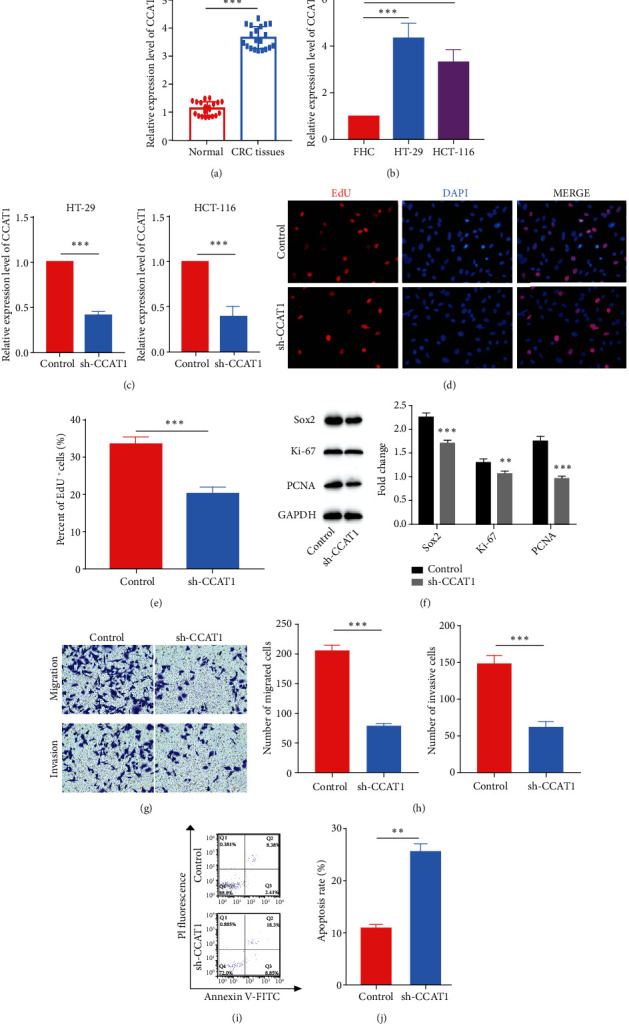
Knockdown of lncRNA CCAT1 inhibited the progression of colorectal cancer progression. (a) Relative expression of CCAT1 in CRC and normal tissues. (b) Relative expression of CCAT1 in FHC, a human colon immortalized cell line, and HT-29 and HCT-116, two colorectal cancer cell lines. (c) Relative expression of CCAT1 in HT-29 and HCT-116 after knockdown of CCAT1. (d) EdU staining was performed to detect the proliferation ability after the knockdown of CCAT1. (e) The statistical result of (d). (f) WB was performed to detect the levels of proliferation markers. (g) Transwell migration and invasion assays were performed to access the migration and invasion abilities after the knockdown of CCAT1. (h) The statistical results of (g). (i) FACS was performed to analyze the apoptosis rate after the knockdown of CCAT1. (j) The statistical result of (i). ^∗∗^*p* < 0.01 and ^∗∗∗^*p* < 0.001.

Firstly, we found that in colorectal cancer tissues, the expression level of lncRNA CCAT1 was remarkably increased (Figure 1(a)). Next, we measured the level of lncRNA CCAT1 in human colon immortalized cell line FHC and two colorectal cancer cell lines, HT-29 and HCT-116, and found that lncRNA CCAT1 was also upregulated in colorectal cancer cell lines (Figure 1(b)). To study the role of lncRNA CCAT1 in the progression of colorectal cancer, we designed shRNA targeting CCAT1 to knock down CCAT1. We transfected cells with sh-CCAT1, measured the expression level of CCAT1 by quantitative PCR, and confirmed the high knockdown efficiency of sh-CCAT1 (Figure 1(c)). We then knocked down CCAT1 in HT-29 cells using sh-CCAT1. CCAT1 knockdown significantly decreased the number of EdU-positive cells and reduced the expression levels of cell proliferation markers, including Sox2, Ki-67, and PCNA (Figures 1(d)–1(f)). Also, we examined whether lncRNA CCAT1 regulated the migration and invasion progresses of CRC cells. Results showed that sh-CCAT1 inhibited the migration and invasion abilities of HT-29 cells (Figures 1(g) and 1(h)). At last, we detected the apoptotic rate of HT-29 cells after CCAT1 knockdown and found that sh-CCAT1 remarkably increased the apoptosis rate (Figures 1(i) and 1(j)). To summarize, knockdown of lncRNA CCAT1 inhibited the progression of colorectal cancer *in vitro*.

### 3.2. hsa-miR-4679 Directly Bound to lncRNA CCAT1

**Figure 2:**
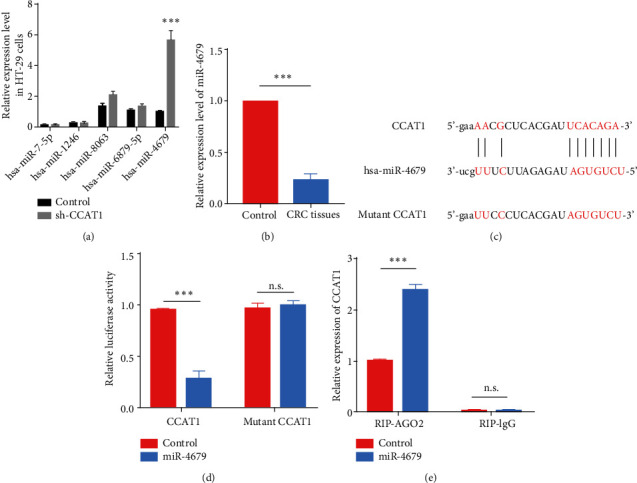
hsa-miR-4679 directly bound to lncRNA CCAT1. (a) The relative expression level of hsa-miR-7-5p, hsa-miR-1246, hsa-miR-8063, hsa-miR-6879-5p, and hsa-miR-4679 after knockdown of CCAT1. (b) The relative expression level of hsa-miR-4679 in CRC and normal tissues. (c) The strategy for the construction of luciferase reporter vectors to detect the binding between CCAT1 and hsa-miR-4679. (d) Relative luciferase activity in cotransfection of CCAT1 and hsa-miR-4679 group was significantly reduced. (e) RIP assay with anti-AGO2 antibody was performed to confirm the binding between CCAT1 and hsa-miR-4679. ^∗∗^*p* < 0.01 and ^∗∗∗^*p* < 0.001.

lncRNAs have been reported in accumulating studies to be involved in the development of colorectal cancer by sponging microRNAs to regulate the expression of downstream proteins. To explore the potential mechanism underlying lncRNA CCAT1 promoting CRC, we conducted bioinformatics analysis using LncBase v.2 and found multiple microRNAs with CCAT1 binding potential, including hsa-miR-7-5p, hsa-miR-1246, hsa-miR-8063, hsa-miR-6879-5p, and hsa-miR-4679, which were previously reported to be abnormally expressed in several cancers [24–28]. We then determined the relative expression levels of these microRNAs and found that hsa-miR-4679 was negatively correlated with CCAT1 level in HT-29 cells (Figure 2(a)). Furthermore, we found that hsa-miR-4679 level was significantly decreased in CRC tissues (Figure 2(b)). To test the direct binding between hsa-miR-4679 and lncRNA CCAT1, we constructed luciferase reporter vectors with lncRNA CCAT1 fragment containing potential hsa-miR-4679 binding site (Figure 2(c)). Cotransfection of CCAT1 and hsa-miR-4679 into HT-29 cells significantly decreased the relative luciferase activity, while cotransfection of mutant CCAT1 and hsa-miR-4679 had no effects on the luciferase activity (Figure 2(d)). Moreover, an RNA immunoprecipitation (RIP) assay was conducted to demonstrate the binding between CCAT1 and miR-4679. The result showed that CCAT1 was significantly enriched by upregulation of miR-4679 with anti-AGO2 in HT-29 cells (Figure 2(e)), indicating the endogenous interaction between CCAT1 and miR-4679, and CCAT1 could be the sponge of miR-4679. Taken together, the results suggested that hsa-miR-4679 was directly bound to lncRNA CCAT1 in CRC tissues.

### 3.3. lncRNA CCAT1 Functioned as an hsa-miR-4679 Sponge

**Figure 3:**
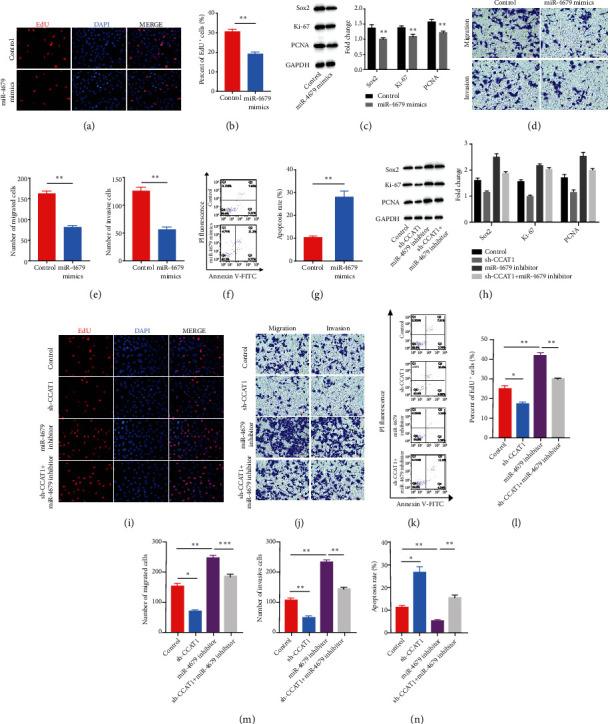
lncRNA CCAT1 functioned as an hsa-miR-4679 sponge. (a) EdU staining was performed to detect the proliferation ability after upregulation of hsa-miR-4679. (b) The statistical result of (a). (c) WB was performed to detect the levels of proliferation markers. (d) Transwell migration and invasion assays were performed to access the migration and invasion abilities after upregulation of hsa-miR-4679. (e) The statistical results of (d). (f) FACS was performed to analyze the apoptosis rate after upregulation of hsa-miR-4679. (g) The statistical results of (f). (h) WB was performed to detect the levels of proliferation markers. (i) EdU staining was performed to detect the proliferation ability after the treatment with sh-CCAT1, hsa-miR-4679 inhibitors, and sh-CCAT1+hsa-miR-4679 inhibitors. (j) Transwell migration and invasion assays were performed to access the migration and invasion abilities after the treatment with sh-CCAT1, hsa-miR-4679 inhibitors, and sh-CCAT1+hsa-miR-4679 inhibitors. (k) FACS was performed to analyze the apoptosis rate after the treatment with sh-CCAT1, hsa-miR-4679 inhibitors, and sh-CCAT1+hsa-miR-4679 inhibitors. (l) The statistical result of (i). (m) The statistical result of (j). (n) The statistical result of (k). ^∗^*p* < 0.05, ^∗∗^*p* < 0.01, and ^∗∗∗^*p* < 0.001.

To verify the functional correlation between lncRNA CCAT1 and hsa-miR-4679, we upregulated hsa-miR-4679 level using hsa-miR-4679 mimics in HT-29 cells. With EdU staining, we found that hsa-miR-4679 mimics significantly decreased the ratio of EdU-positive cells (Figures 3(a) and 3(b)). Proliferation markers, including Sox2, Ki-67, and PCNA, were all downregulated after using hsa-miR-4679 mimics (Figure 3(c)). Similar to the sh-CCAT1 phenotype on migration and invasion of colorectal cancer cells, hsa-miR-4679 mimics hindered the migration and invasion abilities (Figures 3(d) and 3(e)). hsa-miR-4679 mimics also promoted the apoptosis rate of HT-29 cells (Figures 3(f) and 3(g)). To further confirm whether lncRNA CCAT1 is functionally bound to hsa-miR-4679, we downregulated hsa-miR-4679 with an hsa-miR-4679 inhibitor in HT-29 cells and found after hsa-miR-4679 inhibition that the proliferation ability was significantly enhanced. And downregulation of CCAT1 reversed the phenotype of hsa-miR-4679 inhibitor on promoting the proliferation ability (Figures 3(h), 3(i), and 3(l)). We also performed transwell migration and invasion assays and found that sh-CCAT1 significantly decreased migration and invasion levels which were enhanced by an hsa-miR-4679 inhibitor (Figures 3(j) and 3(m)). With the detection of apoptosis rate using PI fluorescence and Annexin V-FITC, we found that sh-CCAT1 also reversed the phenotype of hsa-miR-4679 inhibitor on promoting apoptosis (Figures 3(k) and 3(n)). All results suggested that lncRNA CCAT1 functioned as an hsa-miR-4679 sponge.

### 3.4. hsa-miR-4679 Regulated the Expression of GNG10

**Figure 4:**
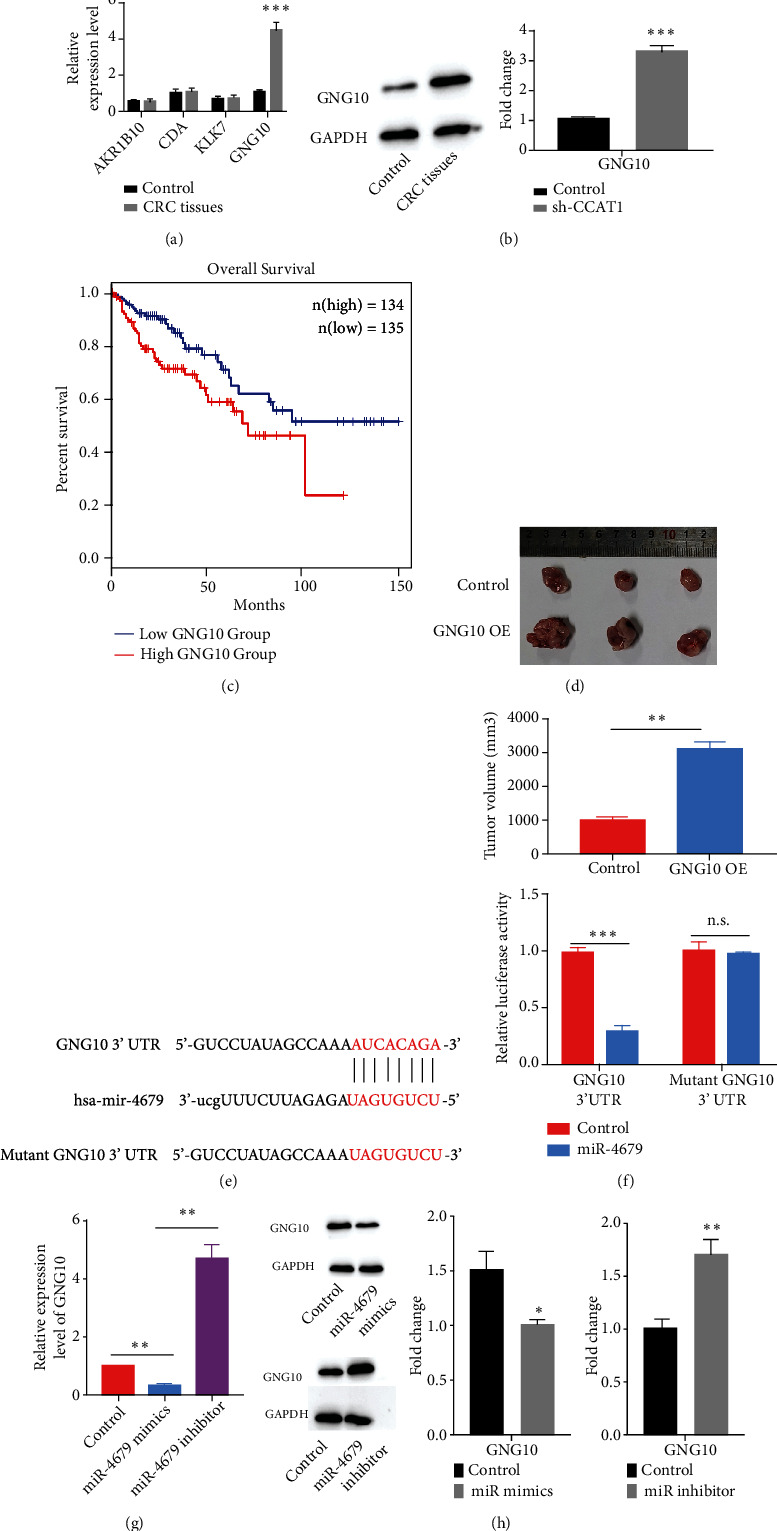
hsa-miR-4679 regulated the expression of GNG10. (a) Relative expression of AKR1B10, CDA, KLK7, and GNG10 in CRC and normal tissues was quantified using qPCR. (b) WB was performed to detect the level of GNG10 in CRC tissues. (c) The expression level of GNG10 was negatively correlated with the overall survival rate. (d) The size of tumor tissues was significantly increased with GNG10 overexpression. (e) The strategy for the construction of luciferase reporter vectors to detect the binding between hsa-miR-4679 and GNG10. (f) Relative luciferase activity in cotransfection of hsa-miR-4679 and GNG10 group was significantly reduced. (g) Relative expression of GNG10 was regulated by hsa-miR-4679 mimics and hsa-miR-4679 inhibitors. (h) WB was performed to detect the level of GNG10 after the treatment of hsa-miR-4679 mimics and hsa-miR-4679 inhibitors. ^∗^*p* < 0.05, ^∗∗^*p* < 0.01, and ^∗∗∗^*p* < 0.001.

lncRNAs could function in a competing endogenous RNA (ceRNA) manner to sponge microRNAs regulating the expression of messenger RNA (mRNA) expression. We performed TargetScan analysis and found multiple binding candidates for hsa-miR-4679, including AKR1B10 (aldo-keto reductase family 1, member B10), CDA (cytidine deaminase), KLK7 (kallikrein-related peptidase 7), and GNG10 (guanine nucleotide-binding protein, gamma 10), which were previously reported to be involved in the progression of cancers [29–32]. GNG10, which was first isolated in 1995, showed only a low level of homology (35-53%) with the other gamma subunits, suggesting the existence of a novel subclass of gamma subunits of the guanine nucleotide-binding protein [33]. Using CRC tissues, we found that the GNG10 level was significantly increased, while the levels of AKR1B10, CDA, and KLK7 were unchanged (Figure 4(a)). Also, the western blot results showed that GNG10 was increased on the protein level (Figure 4(b)). To explore whether GNG10 expression level was correlated with patient survival, we conducted an analysis using the GEPIA2 database (http://gepia2.cancer-pku.cn/#index) and found that the expression level of GNG10 was negatively correlated with the overall survival rate (Figure 4(c)). Next, to further confirm the role of GNG10 on the regulation of CRC progression, HT-29 with stable overexpression of GNG10 was subcutaneously injected into the nude mice. The results showed that GNG10 overexpression significantly promoted tumor growth (Figure 4(d)). Next, we constructed a luciferase reporter system by cloning GNG10 3′UTR into the luciferase reporter vector (Figure 4(e)). We found that cotransfection of vectors containing GNG10 3′UTR and hsa-miR-4679 significantly reduced the relative luciferase activity, while cotransfection of mutant GNG10 3′UTR and hsa-miR-4679 had no effects on the luciferase activity (Figure 4(f)). On both mRNA and protein levels, hsa-miR-4679 mimics suppressed GNG10 level, and hsa-miR-4679 inhibitor increased GNG10 expression (Figures 4(g) and 4(h)). In summary, the expression of GNG10 was regulated by hsa-miR-4679.

### 3.5. hsa-miR-4679 Reversed the Phenotype of GNG10 on Promoting CRC

**Figure 5:**
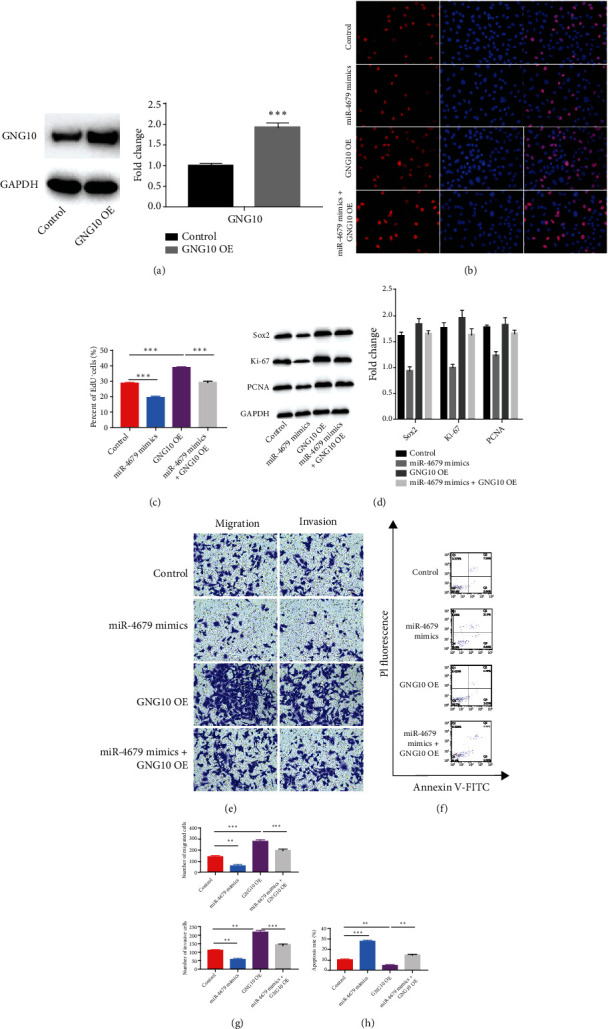
hsa-miR-4679 reversed the phenotype of GNG10 on promoting CRC. (a) WB was performed to detect the level of GNG10 after GNG10 overexpression. (b) EdU staining was performed to detect the proliferation ability after the treatment with hsa-miR-4679 mimics, GNG10 overexpression, and hsa-miR-4679 mimics+GNG10 overexpression. (c) The statistical result of (b). (d) WB was performed to detect the levels of proliferation markers. (e) Transwell migration and invasion assays were performed to access the migration and invasion abilities after the treatment with hsa-miR-4679 mimics, GNG10 overexpression, and hsa-miR-4679 mimics+GNG10 overexpression. (f) FACS was performed to analyze the apoptosis rate after the treatment with hsa-miR-4679 mimics, GNG10 overexpression, and hsa-miR-4679 mimics+GNG10 overexpression. (g) The statistical results of (e). (h) The statistical results of (f). ^∗∗^*p* < 0.01 and ^∗∗∗^*p* < 0.001.

To overexpress the GNG10, we subcloned the GNG10 sequence after the CMV promoter of the pcDNA3.1 vector. Firstly, to confirm whether GNG10 was indeed upregulated after overexpression of GNG10, we measured GNG10 level after GNG10 overexpression. The WB result showed that the GNG10 level was significantly upregulated after GNG10 overexpression (Figure 5(a)). For GNG10 level was upregulated in CRC tissues, we next confirmed the role of GNG10 on promoting CRC by overexpression of GNG10 in HT-29 cells. EdU labeling results showed that GNG10 overexpression increased the ratio of EdU-positive cells, and hsa-miR-4679 mimics reversed the increased EdU-positive ratio (Figures 5(b) and 5(c)). Also, GNG10 overexpression increased the level of proliferation markers, and the increased proliferation ability was reversed by hsa-miR-4679 mimics (Figure 5(d)). To investigate whether hsa-miR-4679 and GNG10 were functionally correlated, we also measured the migration and invasion ability of HT-29 cells in the hsa-miR-4679 mimics+GNG10 overexpression group. The data showed that GNG10 enhanced the migration and invasion of HT-29 cells, and this phenotype was reversed by hsa-miR-4679 mimics (Figures 5(e) and 5(g)). At last, we also found that hsa-miR-4679 mimics enhanced the apoptosis ratio which was decreased by GNG10 overexpression (Figures 5(f) and 5(h)). To summarize, the phenotype of GNG10 on promoting CRC was reversed by hsa-miR-4679, indicating that hsa-miR-4679 regulated GNG10 functionally.

### 3.6. sh-CCAT1 Inhibited the CRC Progression *In Vivo* via the miR-4679/GNG10 Axis

**Figure 6:**
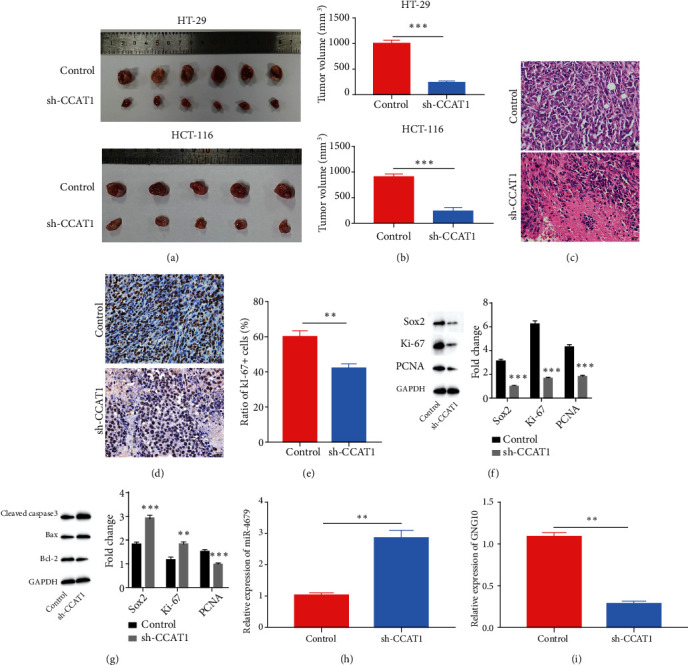
sh-CCAT1 inhibited the CRC progression in vivo via the miR-4679/GNG10 axis. (a) The size of tumor tissues was significantly decreased after sh-CCAT1 using HT-29 and HCT-116 cells. (b) The statistical result of the tumor volume. (c) H&E staining was performed to detect the damages after the treatment with sh-CCAT1. (d) Ki-67 staining was performed to detect the proliferation status of the tissues after sh-CCAT1. (e) The ratio of Ki-67-positive cells. (f) WB was performed to detect the levels of proliferation markers. (g) WB was performed to detect the levels of pro- and antiapoptotic proteins. (h) Relative expression of hsa-miR-4679 after sh-CCAT1 *in vivo*. (i) Relative expression of GNG10 after sh-CCAT1 *in vivo*. ^∗∗^*p* < 0.01 and ^∗∗∗^*p* < 0.001.

To verify whether sh-CCAT1 inhibited the CRC progression *in vivo*, HT-29 cells with sh-CCAT1 were subcutaneously injected into nude mice. Three weeks after the injection, we collected the tumor tissues and measured the tumor size. Data showed that sh-CCAT1 significantly decreased the CRC tumor volume (Figures 6(a) and 6(b)). To further confirm the role of sh-CCAT1 in CRC progression, HCT-116 with stable expression of sh-CCAT1 or the corresponding control was subcutaneously injected into the nude mice. After collecting the tumor tissues and measuring the tumor size, we found that sh-CCAT1 remarkably reduced the tumor size (Figures 6(a) and 6(b)) Furthermore, H&E staining and Ki-67 IHC results revealed that knockdown of CCAT1 significantly reduced the lesion number in the tumor tissues and decreased the Ki-67 proliferation index (Figures 6(c)–6(e)). Also, we measured the level of proliferation marker proteins and apoptosis-related proteins by western blotting. We found that after downregulation of CCAT1 *in vivo*, the levels of proliferation markers, including Sox2, Ki-67, and PCNA, were decreased (Figure 6(f)). Also, sh-CCAT1 promoted apoptosis in vivo by upregulation of proapoptotic proteins (cleaved caspase-3, Bax2) and downregulation of antiapoptotic protein Bcl-2 (Figure 6(g)). To verify whether sh-CCAT1 inhibited the CRC progression *in vivo* via the miR-4679/GNG10 axis, we measured the expression levels of hsa-miR-4679 and GNG10. We confirmed that in sh-CCAT1 CRC tissues, hsa-miR-4679 was upregulated and GNG10 was downregulated (Figures 6(h) and 6(i)). In sum, the results suggested that sh-CCAT1 inhibited the CRC progression *in vivo* via the miR-4679/GNG10 axis.

## 4. Discussion

We have identified a new CCAT1/hsa-miR-4679/GNG10 axis involved in the development of colorectal cancer. We found that CCAT1 was upregulated in CRC, and knockdown of CCAT1 halted the progression of CRC. Next, we found that the expression level of hsa-miR-4679 was negatively correlated with CCAT1, and further luciferase and RIP assays revealed that CCAT1 was directly bound to hsa-miR-4679 in an AGO2 manner. Functional analyses showed that upregulation of hsa-miR-4679 inhibited CRC progression, in which downregulation of hsa-miR-4679 promoted CRC progression by promoting cell proliferation, migration, and invasion and inhibiting cell apoptosis. Also, we found and confirmed the downstream protein effector GNG10, which was regulated by hsa-miR-4679. In the end, we confirmed that sh-CCAT1 inhibited CRC progression via the hsa-miR-4679/GNG10 axis.

CCAT1 has been found to play important roles during the development of several types of cancer, such as gastric carcinoma, colon cancer, gallbladder cancer, and hepatocellular carcinoma, and to affect many biological processes in cancer progression, including the proliferation, migration, invasion, and apoptosis of cancer cells [14–23]. Accumulating studies had revealed multiple molecules function together with CCAT1 during the progression of CRC [20–23, 34–37]. However, the complex regulatory network of CRC development is still poorly understood. Except for previous identified downstream molecules of CCAT1, such as miR-181a-5p, miR-181b-5p, miR-218, and hsa-miR-4679, we identified in this study that there will be more microRNAs involved in the regulation of CRC progression.

Guanine nucleotide-binding protein, gamma 10, which was isolated in 1995, showed a low level of homology with the other subunits, with most of the differences in the N-terminal, suggesting that gamma 10 may interact with a unique alpha subclass [33]. In 2010, GNG10 mutations were found in melanoma, indicating the mutated hetero-trimetric G proteins were involved in melanoma progression [38]. Recently, a study reported that GNG10 was dysregulated and associated with survival of head and neck squamous cell carcinoma (HNSCC) patients [32]. In this study, we revealed that CCAT1 and GNG10 played an important role in CRC progression, providing new potential molecular targets for CRC treatment and drug development. Molecules, including lncRNAs and protein-coding genes, which played significant roles during tumorigenesis, could be promising targets for treating cancer. In cancer treatment, some efforts have been paid for gene therapy with multiple methods for targeting lncRNAs. Antisense oligonucleotides (ASOs) have been clinically used for targeting mRNA to treat cancer [39]. Also, CRISPR/Cas9 genome editing and RNA interference (RNAi) were also useful methods to target lncRNAs [40]. Adeno-associated viruses (AAVs) were an efficient gene delivery system for the nonpathogenicity and its stability within live cells [41]. Because of the lack of knowledge of the precise molecular network regulating CRC, the curative effect of the manipulation of one certain molecule *in vivo* may be still unsatisfactory. Previously, traditional transcriptome analyses of lncRNAs were based on bulk RNA-seq, masking the intratumoral heterogeneity of tumor cells. Long noncoding RNAs, which are polyadenylated, could be captured by poly(dT) oligos and detected by 10x genomics single-cell RNA sequencing. Recently, long noncoding RNAs involved in the development and progression of glioblastoma were identified [42, 43]. To further identify more candidates involved in CRC, transcriptome analyses using patient tissues in single-cell resolution will be useful to identify abnormally expressed noncoding and coding RNAs in a certain cell type.

## 5. Conclusion

In summary, the present study revealed that CCAT1 promoted the progression of CRC and suppressed the expression of hsa-miR-4679. CCAT1 could also regulate GNG10 level via sponging hsa-miR-4679 *in vitro* and *in vivo*. The data suggested that knockdown of lncRNA CCAT1 inhibits the progression of colorectal cancer via hsa-miR-4679 mediating the expression of GNG10.

## Data Availability

All data used in this manuscript are shown in the main figures.
